# A Benchmark Evaluation of Chemical Structure Extraction from Patents: Insights and Challenges in Chemical Structure Recognition

**DOI:** 10.1021/acs.chemrestox.6c00057

**Published:** 2026-05-26

**Authors:** Farina Tariq, Erik Ylipää, Lili Jiang, Patrik Ryden, Patrik L. Andersson

**Affiliations:** † Department of Chemistry, 8075 Umeå University, 901 87 Umea Sweden; ‡ Department of Technology and Natural Sciences (ITN), 4566 Linkoping University, Linkoping 581 83, Sweden; § Department of Computing Science, Umeå University, 901 87 Umea, Sweden; ∥ Department of Mathematics and Mathematical Statistics, Umeå University, 901 87 Umea, Sweden

## Abstract

Early warning systems (EWSs) are currently being developed by various authorities aiming at identifying potentially hazardous chemicals before they become a threat to the environment and human health. In this context, patents provide an excellent data source for exploring novel chemistry or the use of chemicals in materials and products. However, analysis of patents is challenging, including unraveling molecular structures presented as graphics depicting various elements, functional groups, and molecular bonds. Our study aims to improve EWS using automated artificial intelligence-based molecular structure recognition methods for encoding these for further hazard analysis. Current structure extraction tools are primarily trained on chemical structures collected from publicly available data sets, and the application of these tools to patent-specific chemical data has received little attention. This paper presents a field study utilizing the three tools Decimer, Molscribe, and Mathpix and assesses their performance in recognizing chemical structures in patents. Two data sets were compiled and curated including (1) diverse organic chemicals and (2) per- and polyfluoroalkyl substances (PFAS). It was revealed that these tools perform well on simpler molecular structures, whereas they struggle with more complex structural features, including repetitive units, cross-bonding, and Markush structures. Furthermore, it was discovered that these tools are extremely sensitive to image artifacts such as noise from lines and dots or distortions. Overcoming these challenges will be critical before implementation in automated EWS and thereby enable screening of patents for rapid and effective identification of potentially hazardous emerging chemicals.

## Graphical abstract

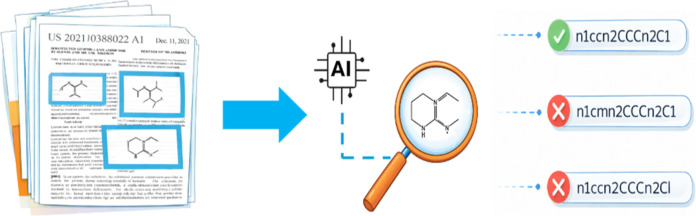

## 1 Introduction

Computational tools^1^ for retrieving chemical information are becoming increasingly important in chemical risk assessment. These tools are essential components of Early Warning System (EWS) designed to detect potentially hazardous chemicals. Many hazardous chemicals are believed to remain undetected, while new, potentially hazardous chemicals are continuously introduced. A critical initial step is to detect novel and Newly Emerging Risk Compounds (NERCs) by systematically searching various data sources, such as scientific databases, literature, and patent documents. Patents are not only legal documents for protecting intellectual property but additionally constitute a huge amount of scientific and technical information.^2^ While many patents might not result in actual products on the market, they can be used to discover novel use of known risk compounds, or new compounds substituting known hazardous compounds which could have similar properties. The “IP5” offices—the EPO (European Patent Office), USPTO (United States Patent and Trademark Office), JPO (Japan Patent Office), KIPO (Korean Intellectual Property Office), and China’s CNIPA (China National Intellectual Property Administration)—oversee examination on a global scale and collectively manage most patent applications.^3^ Espacenet^4^ (run by the EPO) is a useful search engine for patent data curation and analytics since it offers free access to over 100 million patents. To place a published patent within a technical taxonomy, the Cooperative Patent Classification (CPC) expanded from the International Patent Classification (IPC) is assigned to signal the concept of the invention.^5^ These CPC codes can be used for consistent retrieval in platforms, such as Espacenet for specific technological domains.

With the latest developments in artificial intelligence (AI), patent data has been utilized in a variety of sectors, such as medicine, cosmetics, and chemistry, to perform data-driven research and identify trends. Deep learning (DL) models such as BERT have been effectively employed in green chemistry and cosmetics for finding sustainability-focused patents and offering insights into environmentally friendly innovations.^6^ In a similar way, natural language processing (NLP) techniques paired with semantic feature extraction have been used to extract and categorize scientific-technical data, such as chemical phenomena, from patent documents.^7^ Furthermore, explainable AI models have been developed to detect novelty in technical claims, providing transparent support for patent evaluation.^8^ These applications highlight AI’s ability to extract important information from patent literature across scientific areas.

While AI-driven developments have significantly facilitated the extraction of information from patents, an immense amount of chemical information remains undetected. Chemical information is presented in a variety of formats in patent filings such as textual descriptions (IUPAC names), tables of data, and graphical representations of molecular structures. However, most of this information is published in nonmachine-readable formats. Chemical names and contextual information are presented either as tables or text in the description and claims sections, while small-molecule structures and reaction schemes are usually embedded as figures in PDFs as either vector drawings or raster scans (such as TIFF). Additionally, there are complex illustrations that deviate from straightforward single-molecule drawings, such as Markush structures having a single generic structure with variations of substituent groups. Apart from the intricacy of generic chemical representations, specific compound classes, such as per- and polyfluoroalkyl substances (PFASs), present unique molecular structure complexities, such as fluorine-rich backbones that may include generalized repeating units. Moreover, patent images vary greatly in terms of their quality. Many older patent documents (for example, certain World Intellectual Property Organization-WIPO or European patents) include low-resolution or scanned chemical drawings. Despite some patent offices, such as the USPTO, having called for applicants to provide chemical structures as machine-readable formats (e.g., MDL molfiles or ChemDraw CDX files) since 2001,^9^ patents rarely incorporate chemical structure files, such as molfiles, CDXML formats, or line notations like SMILES and InChI. In cases where patents are published as full-text XML, those schemas capture text and bibliographic information but typically do not encode a chemical structure. Thus, a chemical structure depicted as a drawing on a patent page cannot be retrieved using a text search. Therefore, it necessitates optical chemical structure recognition (OCSR) for images to create machine-readable formats that are appropriate for search and analysis.

To effectively extract chemical structures from patents, optical recognition techniques are required to translate these images into standardized chemical representations such as SMILES or InChI strings. Despite large advancements from traditional rule-based systems such as CliDE,^10^ OSRA,^11^ and MolVec,^12^ OCSR is still challenging on real-world patent graphics. Recent transformer-based methods have received interest in the field, one example being Img2Mol,^13^ which uses deep encoders to map images to SMILES, MolScribe,^14^ which uses OCSR for image-to-graph generation, DECIMER^15^/DECIMER.ai,^16^ which combines segmentation, classification, and a transformer OCSR engine, and SwinOCSR,^17^ which uses a Swin Transformer backbone. These concepts are extended to diagram parsing for reaction schemes by RxnScribe^18^ and ReactionDataExtractor.^19^ In addition to these tools, the commercial tool Mathpix^20^ provides image-to-SMILES (and MOL/InChI) conversion for printed and even handwritten diagrams. Prior studies have shown that deep-learning-based systems typically outperform conventional rule-based approaches when comparing the accuracy of modern OCSR tools on generic patent data sets.^21^ However, the behavior of OCSR tools on highly specialized chemical domains and their potential to be incorporated into an EWS aimed at monitoring chemical innovation through patent documents have not been addressed by these evaluations.

This study aimed to investigate current AI-driven methods to translate chemical structures in patent documents to machine-readable formats and to support their integration into an EWS. For this purpose, we tested three OCSR tools including DECIMER, MolScribe, and Mathpix on two curated patent data sets, here termed (1) the general organic chemistry (GOC) data set and (2) the per- and polyfluoroalkyl substances (PFAS) data set. Rather than focusing solely on overall accuracy, we aimed to identify strengths and weaknesses of these tools in terms of pre/postprocessing workflow and algorithmic improvements, to enable trustworthy implementation in the EWS framework and larger computational screening pipelines.

## 2 Material and Methods

To assess the effectiveness of chemical structure recognition tools in the context of patent data, this study was established around a three-stage research framework (Figure 1). In step (I), a data set of images of chemical structures was collected from chemical patent documents using data curation methods (Section 2.1). In the next step (II), the three selected tools for chemical structure recognition, including an open-source and a commercial model, were used to extract the corresponding SMILES representations (Section 2.2). In the final step (III), the extracted SMILES were examined, and the tools were validated through an expert-based validation to ensure a well-founded assessment of the strengths and limitations of each tool (Section 2.3).

**Figure 1:**
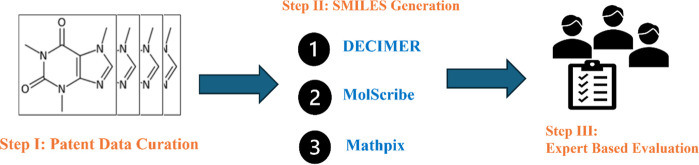
Three-stage framework illustrating (I) Data Curation from the European Patent Office, (II) SMILES Generation using MolScribe, Mathpix, and DECIMER, and (III) expert-based evaluation of generated SMILES.

### 2.1 Data Set Curation

To capture the structural complexity commonly found in chemical and environmental research, the first phase of this study involved curating chemical structure image data sets from patent documents. We used the Espacenet^22^ platform to retrieve patent data of the EPO. To find patents that contain molecular structures, the search profile was based on selected IPC/CPC codes, chemical keywords including “chemical structure” or “molecular structure,” and temporal filters. Two separate data sets were curated entitled GOC set and PFAS set. For the GOC set, C07C code patents were searched during the time period January 2021 to May 2024 using keywords in the title, abstract, and claims fields. For the PFAS set, Espacenet query included the presence of fluorine-related terms (e.g., “fluorinated”, “PFAS”), classification codes under Section C (for chemistry), and keywords appearing in the abstract, claims, or title. In the next phase of the data curation, a manual image cropping was performed to extract chemical structure images embedded in multipage PDF documents. It should be noted that preprocessing techniques were not applied on the raw images as the goal was to evaluate the performance of the selected tools in genuine, real-world scenarios rather than masking the inherent limits in practice via preprocessing techniques.

### 2.2 SMILES Generation

Three OCSR tools were chosen for the extraction of chemical structures from images: (i) DECIMER^15^ (Deep Learning for Chemical Image Recognition) is a neural network-based tool trained on large data sets of synthetic chemical images, and it converts chemical structure diagrams into SMILES strings, (ii) MolScribe^14^ is a transformer-based molecular optical recognition model that is trained to understand complicated and low-quality chemical structure images, and (iii) Mathpix^20^ is a general-purpose commercial optical recognition engine that can retrieve LaTeX text, chemical equations, and molecular drawings from PDFs and images providing structure information different chemical markup formats, such as SMILES and InChIKey. DECIMER v2.7.1 and MolScribe v1.1.1 were set up and installed according to the official instructions found in the publications or documentation that accompany it. For MolScribe, the pretrained model *swin_base_char_aux_1m* (available via Hugging Face) was employed, whereas a standard pretrained model (available via Zenodo) had been used for DECIMER. The study was carried out on a standard desktop workstation with an Intel CoreTM i7-1265U CPU (1.80 GHz) and 64.0 GB of installed RAM running a 64 bit operating system. Further, the generated SMILES representations were then validated.

### 2.3 Expert-Based Evaluation

To assess the accuracy of generated SMILES from chemical structures in patents, an expert-based validation approach was developed. First, the generated SMILES were converted to structures using RDKit^23^ (v2024.9.6) as the primary transformation tool and CACTUS/CIR^24^ (Web site access via REST API) as the fallback tool. Domain experts (five in total) were presented with the input patent image and three prospective depictions (MolScribe, DECIMER, and Mathpix) and asked to decide if the structures matched per software or if all failed (“no match”). The validation metric was accuracy, which was defined as the ratio of correctly matched images to the total number of evaluated images.

$$Accuracy=\frac{Number\;of\;correctly\;classified\;images}{Total\;number\;of\;images}$$

The validation was carried out in two phases. In the pilot phase, a 20% random sample of the total curated data set was chosen and provided to three domain experts for manual review. Following that, the results were interpreted jointly with all three experts in an organized discussion session. This process indicated that the experts needed detailed common instructions on how to interpret chemical concepts and terminology used in this context including interpretation of chemical abbreviations, handling of rotatable bonds, and stoichiometric arrangement (see Supporting Information). In the full validation phase, the whole data set was validated by a group of five experts that each validated one-fifth. The experts were asked to assess the correctness of output structures by comparing them to the input images using an established set of evaluation criteria specified in the instruction file. The responses from the expert validation were examined for various subgroups of images and the analysis tools.

## 3 Results and Discussion

### 3.1 Data Curation Outcomes

For the GOC data set, 352 chemical structures were retrieved from 88 patents having chemical structure images (Table 1). Several duplicates were identified, resulting in 309 unique chemical structures. In the PFAS data set, retrieved patents yielded 48 chemical structures, and among these structures, 43 were unique. Overall, only a small percentage of the identified patent files of both data sets included chemical structure images, i.e., less than 7% of the GOC data set and only 2–3% of the PFAS set.

**Table 1:** Summary of Collected Datasets (GOC and PFAS) Including Number of Retrieved Patents, Patents Containing Chemical Structure Images, The Total Extracted Chemical Structures, and the Number of Unique Identified Structures

data sets	retrieved patents	patents with structures	chemical structures	unique chemical structures
GOC	1371	88	352	309
PFAS	1075	26	48	43

An analysis of the CPC codes of the hits revealed that while the structure search was tailored on the C07C class (acyclic or carbocyclic compounds), identified chemical structures were also classified in other CPC classes. Among the 500 CPC codes (Table S1), C07C made up 275 (55%) hits, whereas the remaining hits were spread among several CPC codes containing patents related to applications associated with agriculture, analytical methods, polymers, and biological sciences. For the PFAS data set (Table S2), C02F dominated (40%), indicating development in the water and sludge treatment area. Additionally, PFAS patents were found in, e.g., G01N, A62D, and B09C, indicating that PFAS are used in applications including chemical analysis, firefighting technology, and handling hazardous waste.

### 3.2 Performance of SMILES Generation Tools

All three tools predicted the structures of the GOC data set with similar performance and accuracy above 74% (Figure 2a). DECIMER showed the highest accuracy, correctly identifying 242 out of 309 images, MolScribe had a 75% accuracy rate, correctly identifying 234 out of 309 images, whereas Mathpix had a 74% accuracy rate, correctly identifying 229 out of 309 images. For the PFAS set, DECIMER and MolScribe performed better than Mathpix, which had much lower accuracy (Figure 2b). A sizable amount of the whole PFAS data set consisting of 26 chemicals did not produce an accurate result from any of the tools, demonstrating the significant limitations of existing OCSR tools for PFAS.

**Figure 2:**
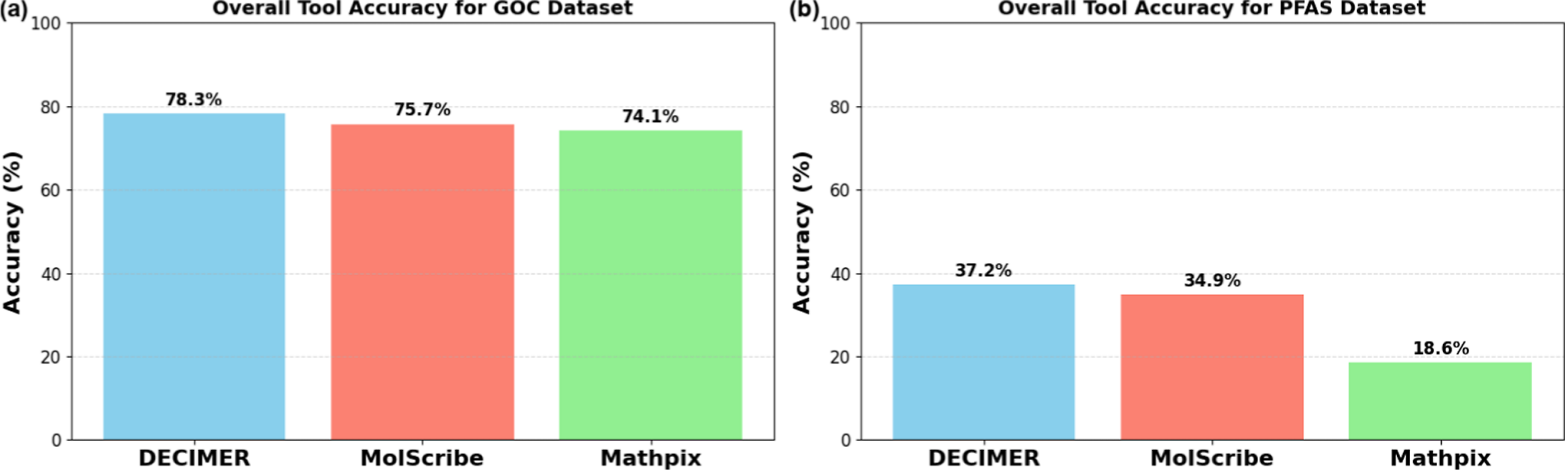
Overall tool accuracy for (a) the GOC data set and (b) the PFAS data set.

### 3.3 Correctly Identified Structures

Figure 3 illustrates a representative set of structures that all three tools correctly recognized. In a nutshell, these examples shared high-quality drawings with standard drawing conventions. All tools were able to decode polycyclic aromatic scaffolds and the linkage -*O*-(CH2)­5–*O*- (Figure 3a). Furthermore, the three tools were able to resolve abbreviations including Bn (−CH_2_–C_6_H_5_), Cbz (−COOCH_2_–C_6_H_5_), Boc (−COOC­(CH_3_)_3_), and OMe = −OCH_3_ as shown in Figure 3b,c. Structures with tertiary-amines and tetravalent silicates were correctly assigned by all tools (Figure 3d,e). Similarly, a subset of structures in the PFAS data set having a perfluorinated carbon chain with carboxyl or sulfonic acid moieties (-OSOOH or –OOH) (Figure 3f) were successfully extracted by all tools irrespective of chain length.

**Figure 3:**
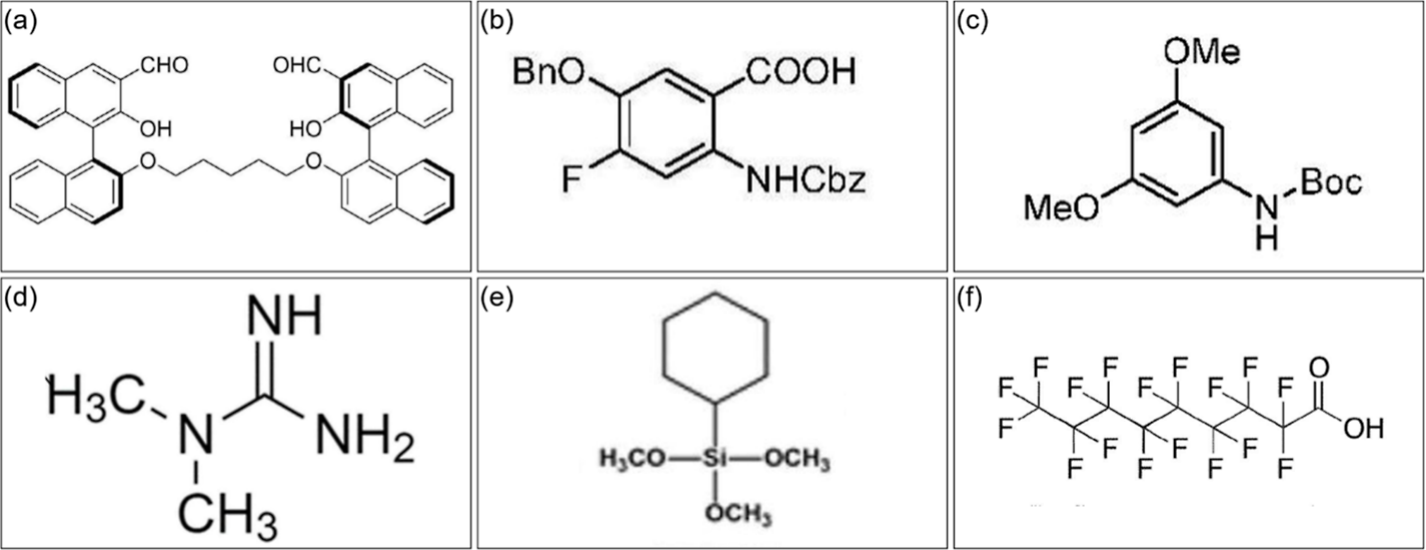
Representative chemical structure images for which all three tools produced correct SMILES predictions.

### 3.4 Poorly Identified Structures

Markush images were challenging where distinct core molecular scaffolds can be substituted with different functional groups identified with R- or X (Figure 4a–c). For these structures, MolScribe kept the molecular backbones and generated SMILES with “*” at the X- and R-positions instead of producing a labeled placeholder (e.g., [X] or [R]) as shown by Figure 4d–f, whereas DECIMER and Mathpix generated SMILES strings with explicitly retained Markush labels (e.g., R, X). But when these SMILES outputs were handled with cheminformatics tools like RDKit and CACTUS for image generation, SMILES parsing issues occurred as unresolved Markush placeholders were not supported by SMILES language. Out of 15 images containing Markush structures, none of the images were classified correctly by any of the tools (Table S3).

**Figure 4:**
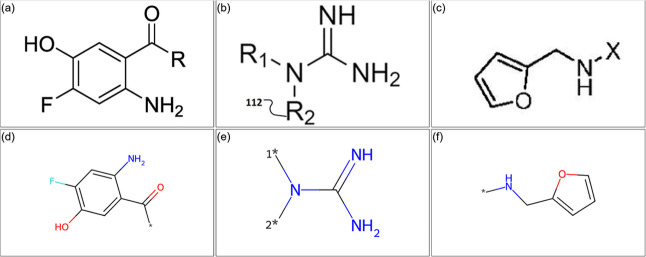
Examples of structures with Markush subgroups with R or X labels where (a–c) is examples of input structures and (d–f) the corresponding output structures using MolScribe.

Table 2 shows two subsets of images that were poorly decoded, including (1) certain abbreviations and (2) compressed or repetitive chemical units. For the abbreviation category including 93 entries, MolScribe outperformed the other two tools with 77 correct predictions, whereas Mathpix and DECIMER resulted in 72 and 62 correct predictions, respectively. The left column shows varied issues with the *tert*-butyl-group where DECIMER correctly assigned the group, whereas the other two methods decoded them erroneously (Figures a,c,e,g). DECIMER on the other hand had issues with the abbreviation Ph_2_P which should be phosphorus with two phenyl groups, which was correctly assigned by the other two methods. The second challenging abbreviation illustrated by structure b in Table 2 is an aryl group (Ar), which was not assigned correctly by any method. The common pattern among these cases was that the cores were mostly identified, but abbreviation sites were either misexpanded, dropped, or created as literal textual tokens (e.g., Ph2 and [Ar]). The two example structures to the right (i,j) illustrate graphics where structural formulas were mixed with molecular formulas to compress large and repetitive parts of the chemical. These two examples are from the GOC and the PFAS set, and clearly, none of the methods were able to decode these shown by Figures k-m. DECIMER was able to partly decode the first structure (i) but with the wrong number of carbons. The errors were limited to the interpretation and expansion of the numeric chain labels, but all of the tools were able to capture the core scaffolds. Thus, the analysis showed that these OCSR tools were not capable of handling compressed notations, whereas DECIMER was more problematic in reading abbreviations (Table S3). The complete stratified analysis of data is available in the project repository via Zenodo (see Data and Code Availability statement).

**Table 2:**
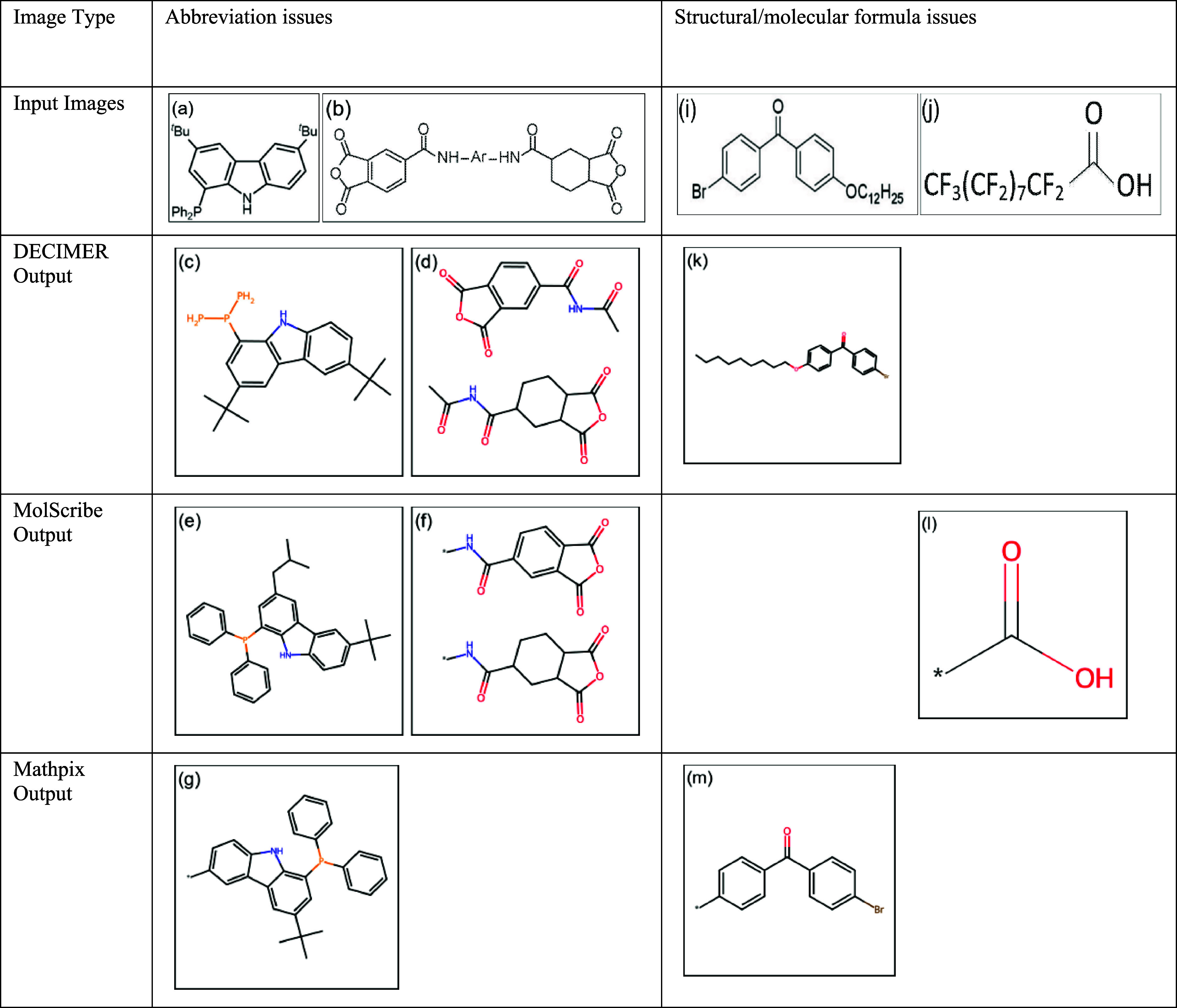
Examples of Two Challenging Types of Images Including the Left Columns of Abbreviation Issues (a, b) and the Right Columns with Compressed/Repetitive Notations (i, j)^a^

a Results from the three OCSR tools—DECIMER, MolScribe, and Mathpix—are shown in the rows below each corresponding input structure.

### 3.5 Discussion

An EWS workflow starts with either a particular triggering signal of an effect or exposure risk or a systematic and thorough data collection and curation.^1^ In the latter case, that could include compiling inventories of environmental and human monitoring data or toxicity data, aiming at identifying potential NERCs after a thorough computational risk screening analysis. Larger collaborative initiatives like the NORMAN network show how early identification of potential NERCs could lead to environmental monitoring, prioritization, and policy support for contaminants of emerging concern (CECs).^25,26^ In this regard, a relevant source of information for EWS is patent data, revealing novel chemicals years before they show up on the market. In patents, chemical structure information can be embedded, and thus, reliable chemical structure recognition tools are needed for an EWS to operate efficiently. However, automated screening of chemicals in patents is still challenging, as structures are included as images rather than in a machine-readable format. In this regard, OCSR tools enable automated extraction of chemical structures from images.

To the best of our knowledge, the existing literature remains limited on benchmarking of OCSR tools in the context of patent mining. The existing research on OCSR tools has mostly focused on benchmarking traditional tools or reviewing the methodological developments. Musazade et al.^27^ offered a thorough analysis of the development of OCSR techniques, highlighting the shift from rule-based pipelines to deep-learning-based models. Similarly, Rajan et al.^28^ offered a review of OCSR tools and evaluated rule-based tools like OSRA, Imago, and MolVec across several public data sets. This benchmark indicated that rule-based tools were more sensitive toward images with lower quality or domain-specific drawing conventions, resulting in lower accuracy, as seen for patents from several offices including JPO and USPTO. Recently, a comprehensive assessment of modern OCSR technologies was completed, where eight algorithms were evaluated on patent images covering different structures and reaction schemes.^21^ This analysis revealed significant performance differences between studied tools based on image content, and they concluded that no recent tool offers the correct extraction of chemical structures. Although their analysis showed that the accuracy of the tools varied significantly depending on the type of image, they did not analyze how these constraints spread to data curation tasks and use in automated workflows, such as EWS. In addition, chemical categories of importance in the area of emerging pollutants, such as PFASs, were not analyzed. Our study is the first to assess these tools in the context of EWS deployment, emphasizing the types of extraction failures that constrain their application for real-time regulatory monitoring.

In line with the results of Krasnov et al.^21^ explained above, all three tools demonstrated comparable performance on standard, well-drawn organic structures. Our data show that the tools can encode aromatic rings, functional groups (either as simple textual abbreviations or full atom-bond subgraphs), and heteroatom valence patterns in accordance with atom typing and order assignment. This indicates a close alignment toward the training data of OCSR tools, which consists of clear, high-quality images of chemical structures. For instance, MolScribe uses patent-derived structures from USPTO Molfiles in addition to randomly chosen PubChem structures generated synthetically for training purposes (see Supporting Information of ref 14). Despite the acknowledged noise, these structures still offer explicit atom and bond information. DECIMER is trained by using hundreds of millions of artificially produced depictions made with a variety of cheminformatics toolkits and substantial image augmentation for reliable performance across a wide range of depiction styles (see Supporting Information of ref 15). Across the two data sets collected, we have observed a significant shift in terms of predictive performance. For the GOC data set, the three analyzed tools are stable and mature except structures having Markush representations, abbreviated functional groups, repeating units, and compressed chemical formulas. For PFASs, all tools struggled with fluorinated chains and showed low predictive performance.

### 3.6 Markush Structure

A fundamental conflict between what the current OCSR pipelines are designed for and how patents frequently express chemical structures is revealed by Markush structures. In these structures, substituents are replaced by X or R to cover families of compounds. Consequently, tools that are designed to adhere to SMILES semantics either create placeholders “*” or parse malformed SMILES with Markush labels. Although these outputs show the location of the structural variability, they are incompatible with cheminformatic toolkits. Since there is not a single ground-truth for Markush structures, these images inherently fall into the “no match” category under a correctness metric designed for concrete structures. So, a Markush-aware pipeline starts from preserving Markush placeholders during structure extraction, followed by extraction of applicable functional groups as substituents from patent text and systematically replacement of Markush placeholders with extracted substituents to create complete and multiple SMILES strings.

### 3.7 Structural/Molecular Formula Units

Entries where a structural formula is mixed with a molecular formula to compress longer repetitive units showed to be challenging as current OCSR pipelines have been tailored to parse explicit atom–bond graphs. In such cases, the structural cores are accurate across tools, but the repetitive units, often in the form of longer carbon chains, are incorrectly assigned because the tools need optical character recognition (OCR) plus domain rules in addition to vision-to-graph. For instance, the under-expansion for DECIMER (Table 2k) (C_12_ → C_9_; C_12_/C_10_ → C_9_/C_10_) indicates that either the expander used a default/nearest template instead of the precise count, or that the OCR/interpretation stage lost digits. When a terminal end is annotated with, e.g., C_10_ or C_12_, the tool must (i) read the text via OCR, (ii) linguistically understand that it as an alkyl chain with a specific length, and (iii) transfer it to the correct chain length at the appropriate attachment point.

### 3.8 Abbreviation and Compressed Notation

Patent structures frequently include abbreviations to replace common substitutes, such as MOM, *t*-Bu, Ph/Ph, and Ar, which must be comprehended and expanded into atom-bond subgraphs. OCSR pipelines are trained to comprehend drawn chemistry but when presented with abbreviations, they require (i) OCR to read the label, (ii) a chemistry dictionary to resolve it, and (iii) an interface model to attach the expanded group to the right atom with the correct valence. This study showed that OCR worked for some labels but failed for others where the expansion/attachment step was applied incorrectly or skipped (Table 2c,d). The use of “*” (Table 2d,f,g) indicates that the label recognition collapsed, so a generic wildcard was used to maintain valence making SMILES syntactically valid, but semantically underspecified. The third type of failure mode is an overexpansion where extra atoms are added for *t*-Bu (Table 2e). In this case, the template used was off by a single unit or the attachment position was incorrectly counted, resulting in minor but chemically significant topological error.

### 3.9 PFAS-Specific Failure Modes

In the case of the PFAS data set, the incorrect atom assignment points to two fundamental flaws in the existing OCSR tools. First, H → F mis-assignments show that the atom-recognition modules are unable to consistently differentiate between “H” and “F” labels with similar shapes in low contrast images. Second, the substitution of a wildcard symbol (such as *) or number for hydrogen atoms implies that its OCR component incorrectly interprets hydrogen as digits or asterisks. Furthermore, the PFAS data set includes older patents as no temporal limitation was set, resulting in relatively noisier graphical representations. Image heterogeneity in the data set is due to a combination of scanned documents, vector illustrations, and different editors, causing noise and font irregularities, leading to confused atom-recognition modules. Another fundamental blind spot is revealed by chain truncation of the perfluorinated repeating units (e.g., -(CF_2_)_7_CF_3_), as shown in Table 2j, indicating that the recognition engines are not set up to identify bracketed repeating syntax. Rather, during image-to-structure conversion, the parentheses and alphanumeric text are probably treated as noise or unsupported tokens by the OCR or textual-analysis components, which remove them completely. Theoretically, a strong OCSR tool could convert “(CF_2_)_*n*_” into an iterative SMILES repetition (such as “. repeat­(n)”) or at least recognize a single “CF_2_” unit. However, none of the tools described here are capable of such expansion in practice.

## 4 Conclusion

To support EWS screening workflows, our study evaluated AI-based OCSR tools for chemical structure extraction from the patent literature into machine-readable structures. We benchmarked the three systems DECIMER, MolScribe, and Mathpix using two data sets of patents collected and curated from EPO, one presenting general organic chemistry and one focusing on PFAS structures. Our findings indicate that current tools perform well on standard organic chemical structures with an accuracy of up to 78%, whereas their performance was low for PFAS chemical structures. These tools struggle when dealing with Markush structures, abbreviation expansions, and combinations of molecular formulas with a chemical structure, as in such cases chain syntax and quality of image are more demanding. Our findings show that several real-world obstacles related to data curation and model constraints must be overcome for integration of OCSR-based tools in EWS to be successful. The analysis provides a useful and realistic analysis of chemical structures in patents, but it is not comprehensive over time, language, region, or scientific domain. Therefore, a direction of future work for similar studies could be expansion of the data set via incorporating multioffice patents from USPTO, JPO, and CNIPA in addition to EPO. In addition, the data sets collected in this study are small and have a pronounced imbalance between the GOC and PFAS data sets. Furthermore, the conversion of SMILES to structural images, as used in the current validation method, may be constrained. While the tools utilized in this study worked reliably for the current data set, future studies may benefit from investigating alternate depiction methods, such as the CDK-based 2D depiction tool.^29^

A practical limitation noted during data search and curation was server rate limits, resulting in restricted batched retrievals. This bottleneck could be overcome with specialized APIs, institutional access agreements, or bulk-download pipelines. In terms of tool deployment, large patent corpora imply scaling API usage and significant processing power, especially GPU acceleration. Notably, patent images vary in terms of quality, resolution, orientation, and file format, and preprocessing techniques were purposefully excluded from the current study. These elements could have a significant impact on OCSR accuracy, particularly for tools that are prone to noise, scaling, or rotation of images. Therefore, a preprocessing approach that standardizes images prior to inference, such as deskewing, scaling, noise reduction, boosting contrast, and cropping of embedded substructures, is necessary for effective deployment within an EWS. This is important as incorrect structures may induce unnecessary risk mitigation action if further modeling of exposure and hazard analysis of erroneous structures indicates a potential NERC. Therefore, EWS should include confidence scoring for OCSR outputs to track and transmit uncertainty into the further modeling phases. In addition, an effective EWS requires an integrated strategy that blends OCR recognition with NLP approaches to collect relevant chemical information present in the text in combination with structure recognition. Overall, our study demonstrates that existing OCSR tools perform well on standardized structures of organic chemical compounds encountered in patents but that further developments are required to enable their inclusion in automated EWSs.

## Supplementary Material



## Data Availability

The data set, code, and implementation details supporting this study are available via Zenodo at: https://doi.org/10.5281/zenodo.19495631

## Notes

Distribution of Cooperative Patent Classification (CPC) sections with occurrence counts and percentages for the General Organic Chemistry (GOC) data set and the Per- and Polyfluoroalkyl Substances (PFAS) data set; expert instruction manual for chemical structure validation; and stratified performance results of Optical Chemical Structure Recognition (OCSR) tools by chemical structure category (PDF)
