# Supplementary material for: A Benchmark Evaluation of Chemical Structure Extraction from Patents: Insights and Challenges in Chemical Structure Recognition
Source: Chem Res Toxicol. 2026 May 26;39(7):1349–56. doi: 10.1021/acs.chemrestox.6c00057 (PMC13390035; doi:10.1021/acs.chemrestox.6c00057)
Supplement: Supplementary file 1 [file tx6c00057_si_001.pdf]

*Supporting Information*

# A Benchmark Evaluation of Chemical Structure Extraction from Patents: Insights and Challenges in Chemical Structure Recognition

*Farina Tariq[1]*, Erik Ylipää[2], Lili Jiang[3], Patrik Ryden[4], Patrik L. Andersson[1]*

[1] Department of Chemistry, Umeå University, Sweden
[2] Department of Technology and Natural Sciences (ITN), Linkoping University, Sweden [3] Department of Computing Science, Umeå University, Sweden
[4] Department of Mathematics and Mathematical Statistics, Umeå University, Sweden

* Corresponding author: farina.tariq@umu.se

*Supporting Information*

# Table of Contents

**Table S1:** CPC Section Distribution with Occurrence and Percentage for General Organic Chemistry (GOC) dataset .......... S3

**Table S2:** CPC Section Distribution with Occurrence and Percentage for the Per- and Polyfluoroalkyl Substances (PFAS) Dataset .......... S5

Instruction Manual for Chemical Structure Validation .......... S6

**Table S3:** Stratified Performance of OCSR Tools by Chemical Structure Category .......... S7

**Table S1:** CPC Section Distribution with Occurrence and Percentage for General Organic Chemistry (GOC) Dataset

| *CPC* | *Code Description* | *Count* | *Percentage* |
|---|---|---|---|
| *C07C* | Acyclic or carbocyclic compounds | 275 | 55 |
| *C07D* | Heterocyclic compounds | 52 | 10.4 |
| *C08G* | Macromolecular compounds | 22 | 4.4 |
| *B01J* | Chemical or physical processes (e.g., catalysts) | 17 | 3.4 |
| *C09K* | Materials for specific uses | 15 | 3.0 |
| *C07B* | General methods of organic chemistry | 10 | 2.0 |
| *H01M* | Electrochemical devices (e.g., batteries, fuel cells) | 9 | 1.8 |
| *A61K* | Medical preparations | 8 | 1.6 |
| *G01N* | Investigation or analysis of materials | 7 | 1.4 |
| *H10K* | Semiconductor devices | 7 | 1.4 |
| *C07F* | Organometallic compounds | 7 | 1.4 |
| *A61P* | Therapeutic activity of compounds | 7 | 1.4 |
| *Y02E* | Technologies for energy efficiency | 7 | 1.4 |
| *C08J* | Working-up of macromolecular substances | 6 | 1.2 |
| *A01N* | Biocides, pest repellents or attractants | 5 | 1.0 |
| *C01B* | Non-metallic elements and their compounds | 5 | 1.0 |
| *B82Y* | Nano-technology | 4 | 0.8 |
| *Y02P* | Climate change mitigation in production | 4 | 0.8 |
| *C08K* | Use of ingredients in macromolecular compositions | 3 | 0.6 |
| *B01D* | Separation processes | 3 | 0.6 |
| *C12N* | Microorganisms or enzymes | 2 | 0.4 |
| *C12P* | Fermentation or enzyme-using processes | 2 | 0.4 |
| *C02F* | Treatment of water, waste water, or sewage | 2 | 0.4 |
| *B29K* | Use of specific materials in shaping plastics | 2 | 0.4 |
| *B33Y* | Additive manufacturing | 2 | 0.4 |
| *B29C* | Shaping or joining of plastics | 2 | 0.4 |
| *C10G* | Cracking hydrocarbons | 2 | 0.4 |
| *C07H* | Sugars; nucleosides; nucleotides | 2 | 0.4 |
| *C08L* | Compositions of macromolecular compounds | 1 | 0.2 |
| *A23B* | Preservation of foods | 1 | 0.2 |
| *G02B* | Optical elements, systems or apparatus | 1 | 0.2 |
| *C07K* | Peptides | 1 | 0.2 |
| *Y02A* | Technologies for adaptation to climate change | 1 | 0.2 |
| *C08H* | Derivatives of macromolecular compounds | 1 | 0.2 |
| *Y02B* | Climate change mitigation in buildings | 1 | 0.2 |
| *G03F* | Photomechanical production of textured surfaces | 1 | 0.2 |
| *C12R* | Indexing scheme for microorganisms | 1 | 0.2 |
| *C12Q* | Measuring or testing processes in microbiology | 1 | 0.2 |
| *Y02C* | $CO_2$ capture or storage | 1 | 0.2 |

**Table S2:** CPC Section Distribution with Occurrence and Percentage for the Per- and Polyfluoroalkyl Substances (PFAS) Dataset

| CPC | Code Description | Count | Percentage |
|---|---|---|---|
| *C07F* | Treatment of water, wastewater, or sewage | 75 | 40 |
| *B01J* | Chemical/physical processes (e.g., catalysts) | 19 | 10 |
| *H01M* | Electrochemical devices (e.g., batteries) | 16 | 8.6 |
| *C08G* | Macromolecular compounds | 12 | 6.4 |
| *B01D* | Separation processes | 10 | 5.4 |
| *A62D* | Chemical means for extinguishing fires, etc. | 8 | 4.3 |
| *C08L* | Compositions of macromolecular compounds | 8 | 4.3 |
| *G01N* | Analytical or investigative methods | 8 | 4.3 |
| *B09C* | Waste treatment, disposal | 5 | 2.6 |
| *C08K* | Use of ingredients in macromolecular compositions | 4 | 2.1 |

# Instruction Manual for Chemical Structure Validation

This form is designed to assess the accuracy of chemical structure recognition tools. You will be presented with an image of a chemical structure, along with three versions of the same image generated by three different tools: **MolScribe, DECIMER, and Mathpix.** Your task is to analyze generated chemical structures and determine if these are correctly represented. If none of the images accurately match the input structure, please select "Null (No match)." We expect the validation to take about 1 to 1.5 hours, ensuring thorough evaluation without excessive time commitment.

## Instructions:

- Examine the input image and study the chemical structure provided. Take note of functional groups, substituents, and any special notation.
- To review the generated options, you will see images generated by the following tools: MolScribe, DECIMER and Mathpix.
- If one or more of the generated images correctly represent the chemical structure, select the corresponding option(s). If none of the images match the input structure, select "Null (No match)."
- If no match is identified or if you encounter any uncertainty during the evaluation of a chemical structure, please document your observations with number. You may record these details in a designated log—either on paper or in a CSV file—to ensure that all ambiguous cases are properly tracked for further review.
- A number of abbreviations are given, see below
- **Ignore Stoichiometry in Bond Arrangement:** Please note that you should not consider the precise stoichiometric arrangement of bonds when making your judgement.

Note that some bonds are rotatable and may be presented differently by the tools despite being identical.

## Abbreviations and Special Groups:

When validating the chemical structures, please consider the following abbreviations and their meanings:

- **D** – Deuterium, an isotope of hydrogen with one additional neutron.
- **Me** – Methyl ($CH_3$).
- **Ph** – Phenyl ring (typically represents $C_6H_5$).
- **Et** – Ethyl ($C_2H_5$).
- **Boc** – tert-Butoxycarbonyl [$(CH_3)_3COCO$–].
- **Bn** – Benzyl (–$CH_2$–$C_6H_5$ or –$CH_2Ph$).
- **tBu** – tert-Butyl [–$C(CH_3)_3$].
- **MOM** – Methoxymethyl [–$CH_2$–O–$CH_3$].
- **Cbz** – Carbobenzyloxy [–$COOCH_2Ph$ or –COO–$CH_2$–$C_6H_5$; note that Ph is equivalent to $C_6H_5$].
- **PMB** – para-Methoxybenzyl [–$CH_2$–$C_6H_4$–$OCH_3$].

- **Ar** – Aryl, representing any aromatic ring. (For example, if a benzene ring or any substituted aromatic system is generated, it will be considered correct.

**Table S3:** Stratified Performance of OCSR Tools by Chemical Structure Category

| *Category* | *Count* | *MolScribe Correct* | *DECIMER Correct* | *Mathpix Correct* |
|---|---|---|---|---|
| *Abbreviation* | 93 | 77 | 62 | 72 |
| *Markush* | 15 | 0 | 0 | 0 |
| *Compressed** | 20 | 0 | 1 | 0 |

*Compressed stands for molecular formula causing issues
